# Quantifying measurement uncertainty in renal transplant biopsy assessment

**DOI:** 10.3389/fneph.2024.1458491

**Published:** 2024-10-15

**Authors:** Xavier Loizeau, Marina Romanchikova, Spencer A. Thomas, Moulham Alsuleman, John O. O. Ayorinde, Gavin J. Pettigrew

**Affiliations:** ^1^ Data Science Department, National Physical Laboratory, Teddington, United Kingdom; ^2^ Department of Surgery, University of Cambridge, Addenbrooke’s Hospital, Cambridge Biomedical Campus, Cambridge, United Kingdom

**Keywords:** pre-transplantation biopsy, Remuzzi score, measurement uncertainty, reproducibility, glomerular adequacy, sensitivity analysis

## Abstract

**Introduction:**

Renal transplant biopsies provide insights into graft health and support decision making. The current evidence on links between biopsy scores and transplant outcomes suggests there may be numerous factors affecting biopsy scores. Here we adopt measurement science approach to investigate the sources of uncertainty in biopsy assessment and suggest techniques to improve its robustness.

**Methods:**

Histological assessments, Remuzzi scores, biopsy processing and clinical variables are obtained from 144 repeat biopsies originating from 16 deceased-donor kidneys. We conducted sensitivity analysis to find the morphometric features with highest discriminating power and studied the dependencies of these features on biopsy and stain type. The analysis results formed a basis for recommendations on reducing the assessment variability.

**Results:**

Most morphometric variables are influenced by the biopsy and stain types. The variables with the highest discriminatory power are sclerotic glomeruli counts, healthy glomeruli counts per unit area, percentages of interstitial fibrosis and tubular atrophy as well as diameter and lumen of the worst artery. A revised glomeruli adequacy score is proposed to improve the robustness of the glomeruli statistics, whereby a minimum of 104 µm^2^ of cortex tissue is recommended to keep type 1 and type 2 error probabilities below 0.15 and 0.2.

**Discussion:**

The findings are transferable to several biopsy scoring systems. We hope that this work will help practitioners to understand the sources of statistical uncertainty and improve the utility of renal biopsy.

## 1 Introduction

Renal transplant biopsy is a valuable tool to obtain insights into the functional and structural health of a donor kidney Williams et al. (1). Evidence suggests that kidney assessment via pre-transplant biopsies can increase the pool of available donor kidneys by including functionally healthy organs from older donors that may have been otherwise discarded Remuzzi et al. (2)Vathsala et al. (3). At the same time, retrospective studies failed to establish correlation between the biopsy scores and the transplant outcomes, and the need for research on improving the practice has been recognised Eccher et al. (4) Chen et al. (5) Jadav et al. (6). Several renal biopsy scoring systems exist that attempt to quantify the health of key structures of the kidney including vasculature, glomeruli, and scarring Solez et al. (7); Racusen et al. (8); Munivenkatappa et al. (9) De Vusser et al. (10); Remuzzi et al. (11).

The renal biopsy is motivated by the assumption that the tissue morphology is a better predictor of graft quality than donor age and clinical history alone. A further assumption is that the biopsy is statistically representative of the entire kidney, and hence the biopsy score reflects the graft health. However, the score may be affected by other factors such as natural variability, tissue preparation, staining, imaging, pathologist’s assessment and scoring method Haas (12)Yong et al. (13) Huang et al. (14).

From the measurement science (metrology) perspective, one would say that the viability of the kidney for transplant is the quantity being measured (measurand) through the biopsy scoring (e.g., Remuzzi) calculation (measurement model) based on the measurement of the key morphological features (observable quantities) JCGM 200 (15). This measurement is marred by the variabilities in biopsy preparation, image acquisition and assessment, morphological variability within the kidney (natural variability), and the fact that biopsy scores are imperfect estimates of kidney health. While there are many efforts to improve the consistency of the imaging process, pathology assessment and biopsy preparation, the quantification of the uncertainty of the scoring process and methods to reduce this uncertainty remain to be addressed. This work takes first steps towards closing this gap using the metrological concepts outlined in the Guide to the expression of Uncertainty in Measurements (GUM) JCGM 104 (16).

Our study uses the clinical and morphometric data of 144 biopsies stemming from 16 kidneys of 12 deceased donors to estimate the inter- and intra- donor variability. Firstly, we identify the most statistically robust morphological features on Whole Slide Images (WSI). Secondly, we investigate how the biopsy processing steps affect the measurement of those features, and thirdly we provide suggestions on reducing the uncertainty of those measurements.

## 2 Methods

**Figure 1:**
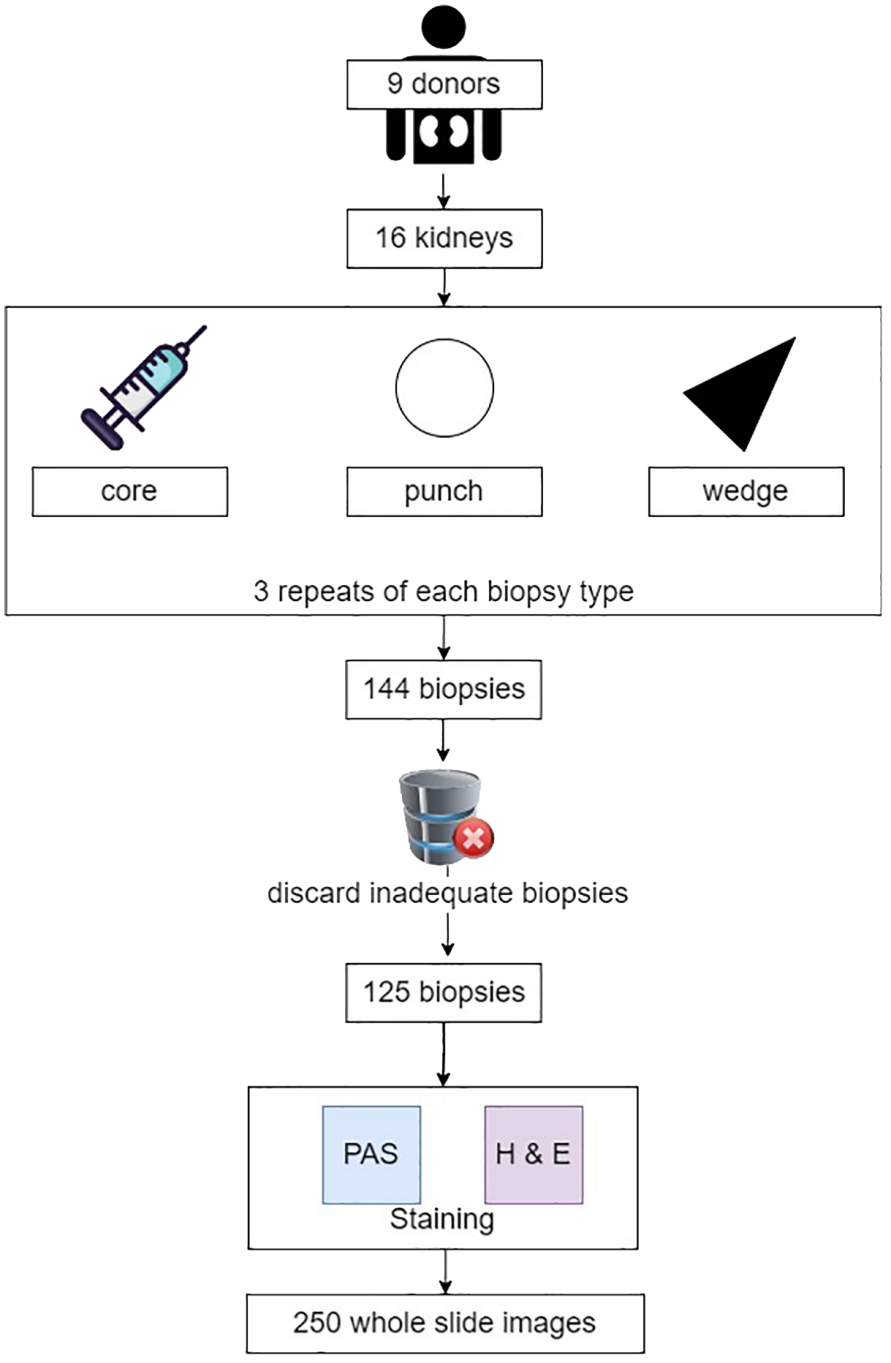
Biopsy acquisition and image selection.

The study aimed to identify the impact of the biopsy processing, morphometric assessment, and intra-patient tissue variability on the measurement of morphological features and the Remuzzi score. Different biopsy retrieval [punch, core and wedge Yong et al. (13)Liapis et al. (17)] and staining techniques [Haematoxylin and Eosin (H&E) and Periodic Acid-Schiff (PAS) Chan (18); McManus (19)] were used to mimic the variations typical in a multi-centre multi-laboratory setting. All biopsies were acquired from 16 discarded kidneys stemming from 12 deceased donors enrolled into the PreImplantation Trial of HIstopathology in renal Allograft (PITHIA) clinical trial Ayorinde et al. (20) Pettigrew (36). The reasons for organ discard were donor quality (*n* = 7), high biopsy score (*n*= 1), suspected malignancy (*n*= 3), sub-optimal perfusion (*n*= 2), renal artery thrombosis resulting from retrieval damage (*n*= 2), and complex cystic lesions (*n*= 1). Punch, core and wedge biopsies were repeated three times on each kidney, yielding 9 biopsies per kidney and 144 biopsies in total. Following a review by a qualified pathologist, 19 biopsies were excluded from the evaluation due to tissue folds or incomplete specimens. Each of the remaining 125 biopsies yielded two histological sections, of which one was stained with H&E and the other PAS stains. 250 WSIs were acquired using a 3DHISTECH Panoramic DESK scanner at 40x magnification with 0.12 µm per pixel. The above process is illustrated in **Figure 1**.

### 2.1 Variable categories

The variables collected for the study were categorised as 1) biopsy processing variables (staining type, biopsy technique, presence of medulla); 2) donor-related variables; 3) morphometric variables. The full list of the variables is provided in **Supplementary Table 2**.

The morphometric variables included cortex area, total, healthy and sclerotic glomeruli [suffering from glomerulosclerosis (GS)] counts, area of Interstitial Fibrosis (IF), area of Tubular Atrophy (TA), wall thickness of the worst artery, lumen diameter of the worst artery, full diameter of the worst artery and presence of medulla on the slide. The worst artery definition is based on the Banff 1997 Working Classification of Renal Allograft Pathology Racusen et al. (8); Lopes et al. (21).

Morphological assessments were performed following semi-blinded and unblinded assessment protocols. In the unblinded protocol, glass slides were assessed on-microscope by an experienced renal histopathologist. In the semi-blinded assessment, digital slides were evaluated by six non-specialist pathologists supervised by an experienced pathologist. To exclude the influence of prior knowledge on the assessment, the slides were evaluated in a random order, whereby one morphometric variable (GS, TA, etc.) was measured at a time.

All assessments for a single kidney were performed by a single pathologist to eliminate the inter-observer variability on organ level.

#### 2.1.1 Derived variables

The morphological assessments described above were used to compute 14 derived variables, including the variables used in the Remuzzi scores (percentages of GS, IF and TA, and wall thickness to lumen ratio), the scores themselves and the transplant decision (single transplant for a score lower than 4, double transplant for a score of 5 or 6 or discard if the score is larger than 7). The variables and the formulae used to calculate them are provided in **Table 1**.

**Table 1:** Intensive and extensive morphometric variables.

Morphometric variable and unit	Computation method	variable type
Biopsy depth and width (µm) Total cortex area (µm^2^) Medulla presence (boolean) Artery puncture (boolean) Sclerotic, and total glomeruli count (count) Healthy glomeruli count (count) Healthy, sclerotic, and total glomeruli per unit area (µm^−2^) Artery, arteriole, and vessel count (count) Artery, arteriole, and vessel per unit area (µm^−2^) Sclerotic glomeruli percentage (%) IF and TA area (µm^2^) IF and TA percentage (%) Vessel and lumen diameter (µm) Wall thickness measured (µm) Wall thickness to lumen diameter ratio (unitless) Remuzzi score, glomeruli ({0,1,2,3}) Remuzzi score, tubular atrophy ({0,1,2,3}) Remuzzi score, interstitial fibrosis ({0,1,2,3}) Remuzzi score, artery ({0,1,2,3}) Total Remuzzi score ({0*,…*,11,12}) Transplant decision (single transplant, dual transplant, discard)	Measured on slide Delineated on slide Identified on slide Identified on slide Counted on slide Glomeruli count −Sclerotic glomeruli count $\frac{\text{Count}}{\text{Total}\;\text{cortex}\;\text{area}}$ Counted on each slide $\frac{\text{Count}}{\text{Total}\;\text{cortex}\;\text{area}}$ $\frac{\text{Sclerotic}\;\text{glomeruli}\;\text{count}}{\text{Total}\;\text{glomeruli}\;\text{count}}\cdot 100$ Delineated on slide $\frac{\text{Area}}{\text{Total}\;\text{cortex}\;\text{area}}\cdot 100$ Measured on the worst artery Measured for the worst artery Computed for the worst artery See **Table 2** See **Table 2** See **Table 2** See **Table 2** See **Table 2** See **Table 2**	Extensive Extensive Intensive Intensive Extensive Extensive Intensive Extensive Intensive Intensive Extensive Intensive Intensive Intensive Intensive Intensive Intensive Intensive Intensive Intensive Intensive

The thresholds for Remuzzi scores used in this work are provided in **Table 2**.

**Table 2:** Threshold values for the four components of the Remuzzi score. Thresholds for IF and TA are identical.

% sclerotic glomeruli	%IF & %TA	vessel wall to lumen ratio	sub-score
[0,2] (2,20] (20,50] (50,100]	[0,6] (6,20] (20,50] (50,100]	[0,0.5] (0.5,0.8] (0.8,1.2] (1.2,∞)	0 1 2 3

#### 2.1.2 Intensive morphometric variables

Area-dependent (extensive) variables such as glomeruli counts were used to derive area-independent intensive variables by dividing them by the kidney cortex area measured on slide. The resulting area-independent intensive variables are denoted as “per unit area”, for example “sclerotic glomeruli counts per unit area”. A list of extensive and intensive morphometric variables and the corresponding computation methods is provided in **Table 1**.

54 of 250 WSIs contained medulla. For those images, cortex area does not coincide with biopsy area. In our dataset, all glomeruli, IF and TA measurements were annotated on the combined cortex and medulla tissues. As medulla area measurements were not acquired, we could not compute intensive variables for these images and refer to them further as “missing values”.

### 2.2 Data analysis

**Figure 2:**
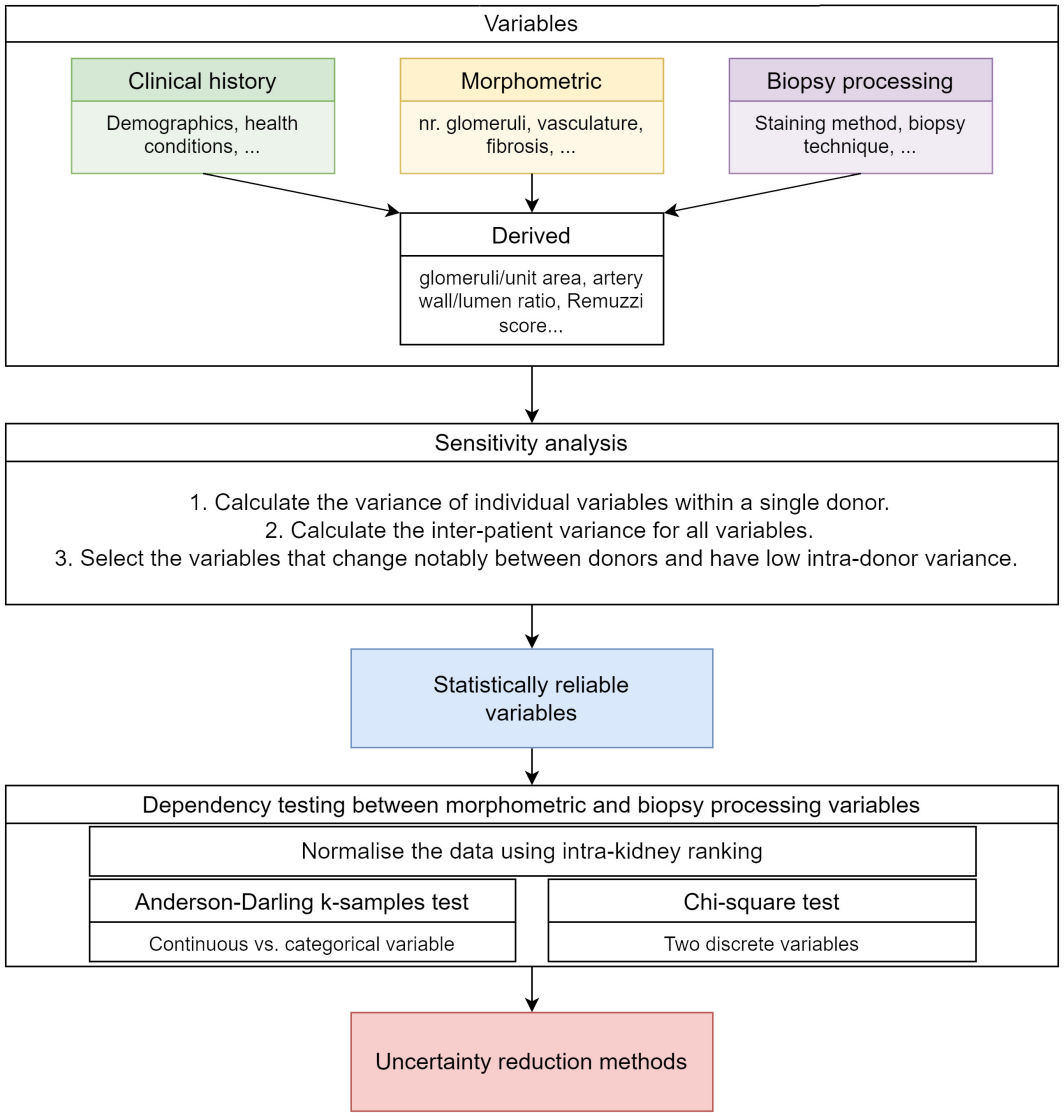
Data processing and analysis steps.

The data analysis followed several stages illustrated in **Figure 2**. Firstly, derived and intensive variables were computed as described in section 2.1. Secondly, the best kidney health indicators (i.e., the variables that show low intra-donor variance and high inter-donor variance) were determined using a sensitivity analysis. Thirdly, dependencies between the biopsy processing steps and the morphometric variables were assessed using statistical testing to determine whether biopsy processing affects the morphological variables measured value. The calculation details are provided in appendix 1.1.3 and section 2.2.1. All calculations and data analysis were implemented using MATLAB version R2022b The MathWorks Inc. (22).

#### 2.2.1 Sensitivity analysis of biopsy morphology to kidney health status

Sensitivity analysis was used to identify the morphometric variables most indicative of inter-donor variations in kidney health. While no data on the kidney functional health was available, one could assume that morphological characteristics of kidneys vary between donors, reflecting individual variabilities and donor clinical history (**Supplementary Table 3**). This inter-donor variability can be used as an estimator of the graft health. We further assume that kidneys from the same donor have similar morphological properties. Using these two assumptions, we obtain the inter- and intra-donor variances and estimate the discriminating power of a variable as the fraction of variance explained by differences between donors. Inter- and intra-donor variance for vessel-related morphometric variables such as wall thickness were calculated using PAS-stained WSI to reflect current clinical practice.

#### 2.2.2 Dependence testing

The impact of biopsy processing methods on morphometric variables was assessed through statistical dependence testing. A ranking process was used prior to the testing to ensure the variations attributed to biopsy processing were not masked by the variations arising from inter-kidney variability. For the ranking, the variable values were grouped by variable and by kidney, ordered and assigned ranks. Rank ties (i.e., when two or more images from a kidney have the same value for a variable) were resolved by assigning an average of two adjacent ranks. In each kidney, the ranks were divided by the number of observations per kidney. An example of rank computation is provided in appendix 1.1.2.

The variable dependencies were tested by performing the χ^2^ test for pairs of categorical variables and the Anderson-Darling k-samples test for continuous/categorical variable pairs Scholz and Stephens (23); Trujillo-Ortiz (24).

In these tests, the null hypothesis is “the two variables are independent”. Rejecting the null hypothesis means that the data suggests that the two variables are dependent with the probability defined by the False Detection Rate (FDR). The tests that reject the null hypothesis indicate that the biopsy processing variable is dependent on the morphometric variable. The tests can be affected by Type 1 and 2 errors. A Type 1 error occurs when two independent variables are declared as dependent (falsely rejecting the null hypothesis). A Type 2 error occurs, conversely, when two dependent variables are declared as independent (falsely accepting the null hypothesis). When multiple tests are run on a set of variable pairs (78 tests), FDR is the proportion of Type 1 errors across all tests, i.e., the proportion of tests that declared two independent variables as dependent. In this work the FDR was kept below 1.5% by using the Benjamini and Hochberg correction Benjamini and Hochberg (25); Williams et al. (26).

### 2.3 Designing an adequacy criterion for glomeruli counts

The fraction of sclerotic glomeruli or percentage of GS is an important marker of kidney health. However, the “true” GS percentage in cortex is unknown, as the glomeruli statistics in the biopsy may not be representative of other cortex regions and are subject to measurement uncertainty. In this work we compute the uncertainty in percentage of GS from first principles for kidneys with varying burden of GS. We use statistical testing theory to compute the minimal number of glomeruli $n^{☆}$ to assess in order to limit the probability of Type 1 (declaring the “true” percentage of GS lower than it is in reality) and Type 2 (declaring the “true” percentage of GS higher than it is in reality) errors to chosen levels. The total number of glomeruli per unit area is collected from 239 (82%) WSIs. Multiplying $n^{☆}$ by the glomeruli density per unit area, we obtain a recommendation for the cortex area required to reduce the uncertainty in glomeruli statistics. Calculation details and theory are provided in appendix 1.1.3.

## 3 Results

**Figure 3:**
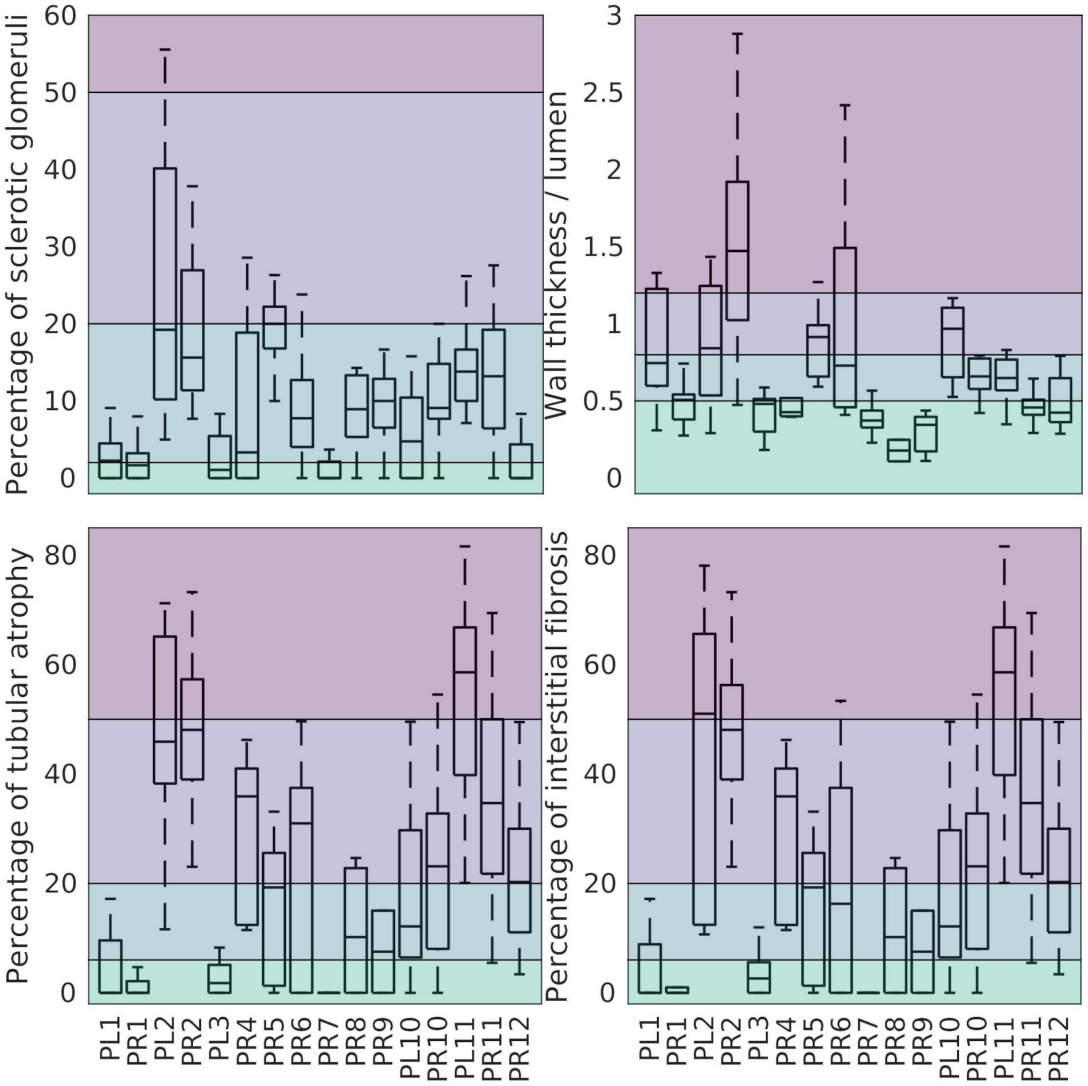
Percentage of GS (top-left), wall thickness to lumen ratio (top-right), percentage of TA (bottom-left), and percentage of IF (bottom-right) grouped by kidney. Left/right Kidneys from the same donor share a number (PL1/PR1). The background colour separates the associated Remuzzi sub-scores ranging from 0 (bottom) to 3 (top).

High intra-donor variability was observed in all morphometric variables in this study, including the four variables contributing to the Remuzzi score (**Figure 3**).

The magnitude and likely sources of this variability are discussed below.

### 3.1 Sensitivity analysis

The aim of sensitivity analysis was to identify the variables that were least affected by intra-donor variability while retaining the ability to discriminate between donors (section 2.2.1), using the latter as an estimator of graft health.

**Table 3** compares the contributions of inter- and intra-donor variabilities to the total variance of morphometric variables and Remuzzi scores.

**Table 3:** Inter- and intra-donor variance of morphometric variables and Remuzzi scores.

variable name	% variance explained by	
	Intra-donor variability	Inter-donor variability
Sclerotic glomeruli count Healthy glomeruli per unit area Sclerotic glomeruli per unit area Percentage of sclerotic glomeruli Glomeruli per unit area Glomeruli count Healthy glomeruli count	54.65 58.66 61.65 65.01 67.43 81.33 81.88	45.35 41.34 38.35 34.99 32.57 18.67 18.12
Percentage of TA Percentage of IF Area of IF Area of TA	41.4 44.11 65.70 68.01	58.60 55.89 34.3 31.99
Lumen of the artery Diameter of the artery Wall thickness of the artery Wall thickness/lumen Artery count	63.82 67.49 71.81 75.63 82.32	36.18 32.51 28.19 24.37 17.68
Total Remuzzi score TA score IF score Artery score Glomeruli score	46.22 47.50 49.58 66.18 71.41	53.78 52.5 50.42 33.82 28.59

The variables are grouped by categories: glomeruli, IF and TA, vessels, Remuzzi scores.

From the variables related to the glomeruli, IF and TA, the percentage of TA (58.60%), the percentage of IF (55.89%), the number of sclerotic glomeruli (45.35%), and the number of healthy glomeruli per unit area (41.34%) have the highest explained variance.

Among the artery-related variables, the lumen and artery diameter show largest fraction of variance explained by inter-donor variability (36.18% and 32.51% respectively).

The total Remuzzi score appears to be a slightly better estimator of kidney health than IF and TA sub-scores alone (53.8% vs. 52.5% and 50.4% of variance explained by inter-donor differences). Notably, the original morphometric variables (percentages of IF and TA) outperform the Remuzzi score and their corresponding sub-scores (58.6% and 55.9% of variance explained by inter-donor variability).

### 3.2 Dependence of morphometric variables and biopsy processing

**Figure 4:**
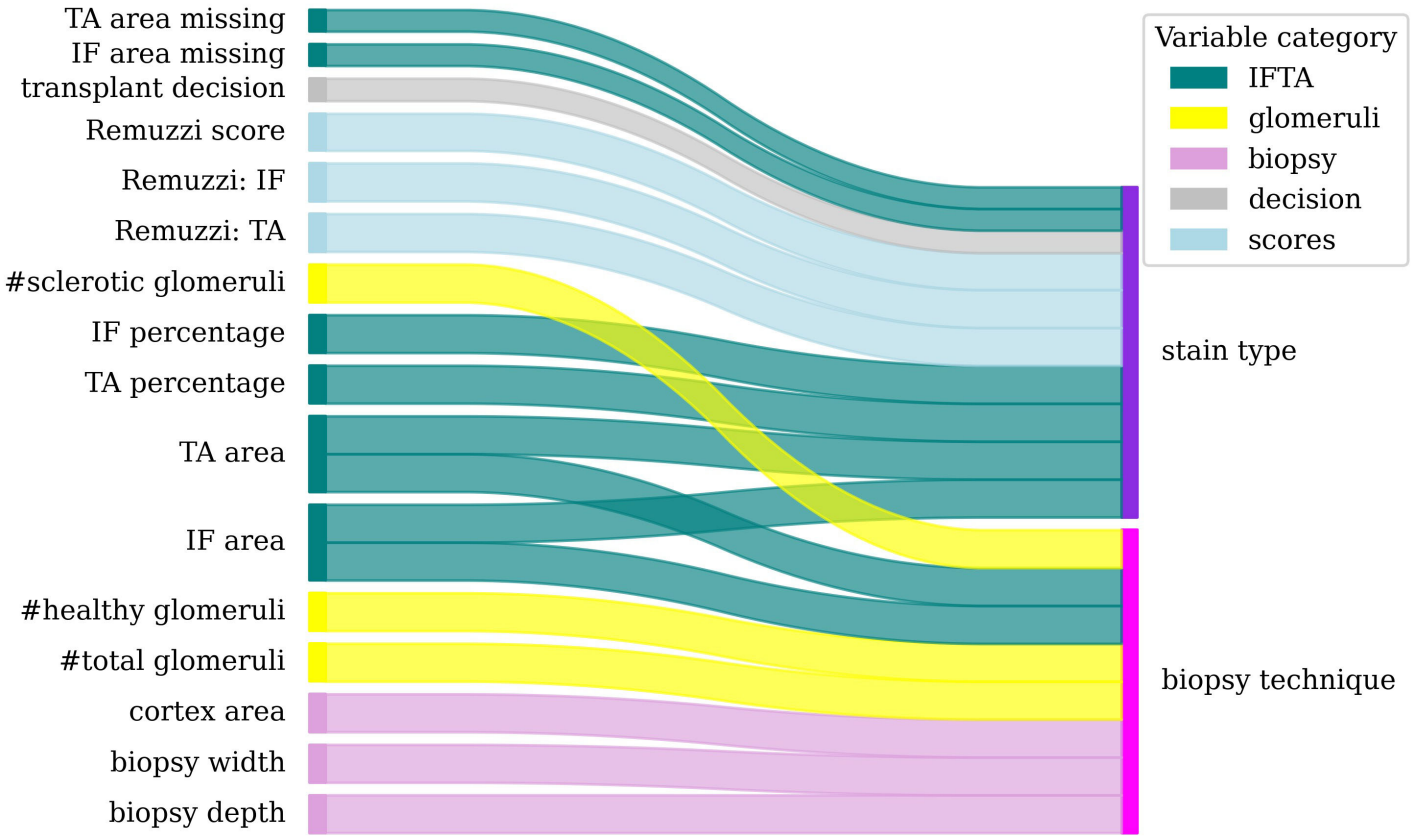
Dependencies between morphometric (left) and biopsy processing (right) variables. Colour band thickness is proportional to statistical significance (*p<*0.001 for all pairs).

78 statistical tests were performed for pairs of morphometric and biopsy processing variables as described in section 2.2.2. The null hypothesis (i.e., there is no link between the variables in the pair) was rejected for 18 variable pairs illustrated in **Figure 4**. All 18 rejected tests had *p* ≤ 0.001 and the expected FDR (in the probabilistic term) is below 0.015.

The tests and the p-values are listed in **Supplementary Table 4**.

The statistical tests revealed the following results. The biopsy technique affected the depth and width of the biopsy, the total cortex area and thus the extensive variables such as glomeruli counts, healthy glomeruli count, area of IF and TA, and sclerotic glomeruli count.

The staining technique was associated with the percentages and areas of TA and IF, the IF and TA scores, the total Remuzzi score, the transplant decision and with the risk of missing values for the area of IF and TA (e.g., when the image did not contain sufficient information to measure these areas).

Overall, PAS staining was associated with higher percentages of IF, and TA in comparison to H&E as shown in **Supplementary Figures 2, 3**.

### 3.3 Adequacy criterion

Statistical testing theory was used to determine the minimum glomeruli count $n^{☆}$ required to limit the probabilities of Type 1 and Type 2 errors (i.e., under- and over-estimating the percentage of GS) to *α* = 0.15 and *β* = 0.2 respectively. The values for *α* and *β* were chosen to provide sufficient confidence in result while minimising a risk of graft injury. The calculation details (and the appropriate way to compute $n^{☆}$ for different values of *α* and *β*) are provided in appendix 1.1.3.

For a biopsy with $n^{☆}$ = 156 glomeruli, if the true percentage of GS exceeds 50, *α <* 0.15, and if GS is ≤ 42.5%, *β <* 0.2. For biopsies where percentage of GS lies between 42.5% and 50%, more glomeruli are required to control the probability of Type 2 error.

**Figure 5:**
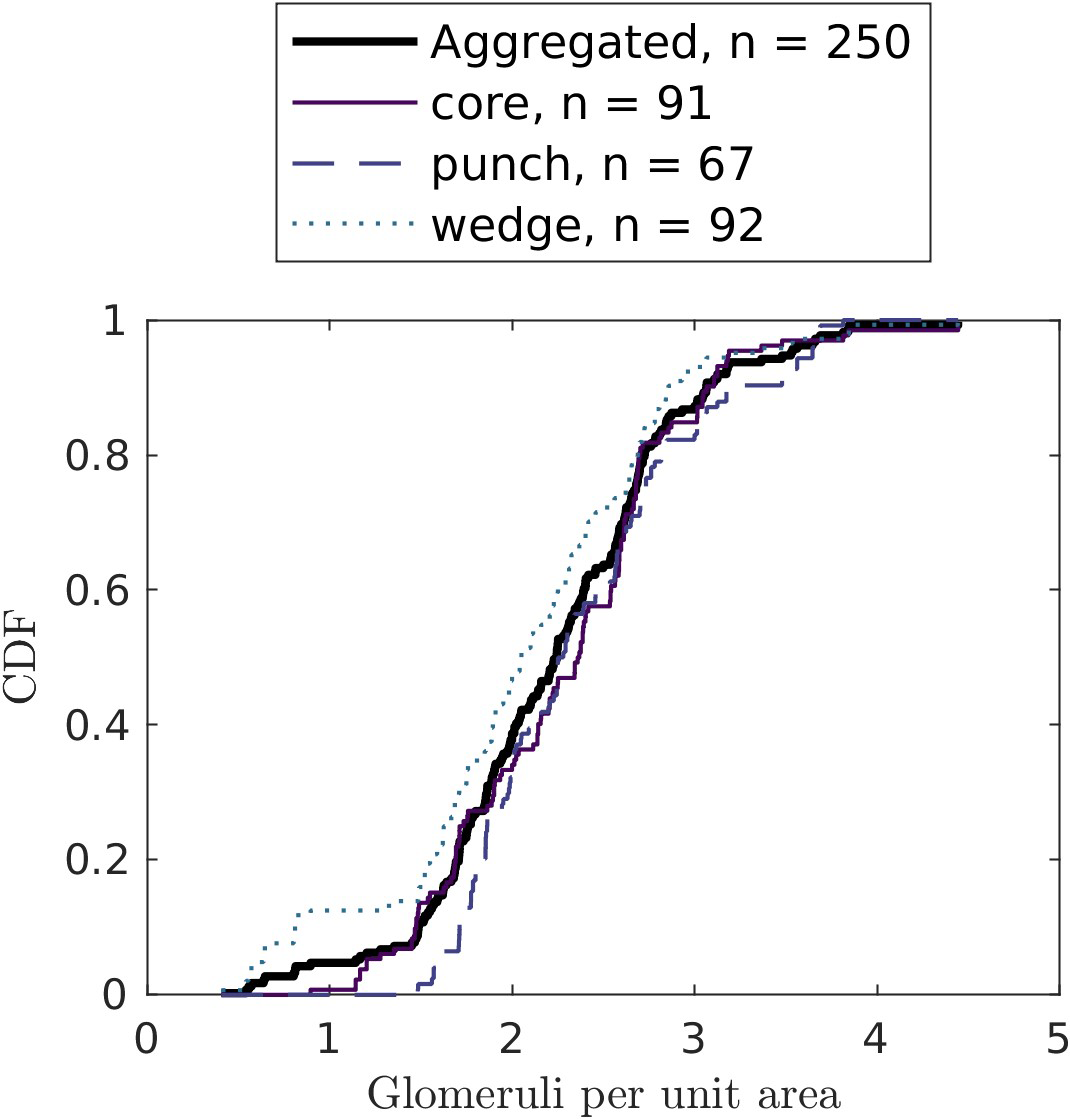
Cumulative distribution functions of glomeruli counts per unit area (in µm^-2^) by biopsy technique. Only the biopsies without medulla (cortex-only biopsies) are considered.

In our sample of 250 images, 90% contained more than 1.5 glomeruli per µm^2^ (**Figure 5**), suggesting that a biopsy surface area of 104µm^2^ has 90% chance to provide enough glomeruli. At the same time, only 16 slides had glomeruli count of ≥ 156, indicating high uncertainties in glomeruli statistics.

## 4 Discussion

This work investigated the factors that may affect the robustness of the common morphometric variables and the resulting Remuzzi scores. As many of the explored variables are used in other scoring systems such as Banff, De Leuven and MAPI Solez et al. (7); Munivenkatappa et al. (9) De Vusser et al. (10), we believe the findings described below will be relevant to many practitioners using renal biopsies.

### 4.1 Statistically robust morphometric variables

The sensitivity analysis (section 3.1) identified six morphometric variables with highest discriminating power, i.e., with low intra-donor variability and high inter-donor variability: 1-2) the percentages of IF and TA; 3-4) the sclerotic glomeruli count and the number of healthy glomeruli per unit area; 5-6) the lumen and diameter of the worst artery. The presence of the sclerotic glomeruli count in the variables with highest variance explained by inter-donor variability is surprising as this is an extensive variable, impacted by biopsy area.

From all morphometric variables, only the percentages of IF and TA were identified as reliable discriminators between donors with 58.6% and 55.6% of variance explained by inter-donor differences. The remaining variability may be attributable to biopsy processing and to tissue non-uniformity within a kidney. Conversion of individual continuous morphological variables (such as percentage of TA) to categorical variables in the Remuzzi score was associated with reduced discriminatory power (that is, decreased fraction of variance explained by inter-donor variability). These results suggest that further work is needed to identify robust morphological variables and to find ways of combining them into a single score, for example by assigning weights to the score components Sethi et al. (27).

As the study was based on the measurements from discarded kidneys and did not have transplant outcome data, inter-donor variability was used as indicator of graft health. Other estimators of graft health such as Kidney Donor Patient Index (KDPI) [Rao et al. (28) Time (29)] could provide additional insights, but the study dataset did not provide the ethnicity information required for KDPI computation. Recent evidence suggests that the KDPI might not be appropriate to define a cut-off in the decision-making process of accepting or declining a kidney graft Dahmen et al. (30) Villanego et al. (31).

### 4.2 Normalisation techniques

Dependency tests showed that the biopsy technique has a strong impact on biopsy area and, consecutively, on the extensive morphometric measurements such as glomeruli counts. We suggest using intensive, e.g., area-normalised variables for the biopsy assessment to account for variations in biopsy surface area.

The association between the staining technique and the measurement of glomeruli, IF, and TA support the practice of using both PAS and H&E stains. In environments with computational pathology facilities, PAS stain could be simulated using virtual staining techniques to reduce the tissue processing efforts in time-critical setting of renal biopsy de Haan et al. (32).

Despite all efforts to control for observer variability and processing all biopsies in the same laboratory, our results demonstrated high intra-patient variability that could be explained by tissue heterogeneity, (for example, due to a presence of localised injury), variations in biopsy preparation (such as stain concentration), or variations in scanner response. Normalisation techniques such as colour calibration should be implemented to minimise these variabilities Ji et al. (33).

### 4.3 Biopsy adequacy

Statistical testing showed that at least 156 glomeruli counts are needed to limit Type 1 (declaring percentage of GS smaller than it is) and Type 2 (declaring percentage of GS larger than it is) errors below 15% and 20% respectively (section 3.3). Only 16 from 250 slides fulfilled this criterion, suggesting that the statistical power of glomeruli counts in renal biopsies may be too low. The insufficient glomeruli statistics may be one of the factors explaining the lack of correlation between renal biopsy scores and transplant outcomes Cockfield et al. (34), where the reported median number of glomeruli per biopsy is 25. This result is consistent with and expands on other recommendations Corwin et al. (35).

We believe that the reliability of renal biopsy measurements can be improved by increasing the biopsy surface area. This increase could be achieved through taking several slices through the biopsy to yield sufficient glomeruli statistics or taking several smaller biopsies from different kidney regions. The time burden associated with the image analysis could be reduced by use of computer-based image analysis tools.

## 5 Conclusions

Morphometric variables and Remuzzi scores are affected by the biopsy processing and evaluation techniques, resulting in large variations in scores and transplant decisions for the same kidney. The sclerotic glomeruli count, number of healthy glomeruli per unit area, the percentages of IF and TA as well as the diameter and lumen of the worst artery had the highest proportion of inter-patient variability and thus were identified as most robust morphometric variables.

With exception of the percentages of IF and TA, intra-donor variability dominated over the inter-donor variability in all morphometric variables, suggesting that biopsy processing and tissue heterogeneity may overshadow the differences between individuals. We believe that the variations introduced by different biopsy processing methods can be accounted for by developing normalisation techniques.

As for the tissue heterogeneities, further research is needed to determine the optimal location of the biopsy to yield most consistent results and improve the biopsy reliability and reproducibility.

We propose a glomerular adequacy criterion of 156 glomeruli to control Type 1 and 2 errors in percentage GS to 15% and 20%. The glomerular density distribution observed in our study suggests that the minimum cortex surface area to yield this number of glomeruli should be 104 µm^2^. Designing similar adequacy criterion for non-uniform tissue regions (section 2.3, appendix 1.1.3) could be useful to improve the GS reliability in presence of heterogeneity. This approach could be adapted to evaluate the surface area requirements for other morphometric variables such as percentage of IF and TA.

Our findings suggest that the reproducibility of morphological measurements in renal biopsies needs improvement. This improvement can be achieved through several measures. Firstly, it is essential to identify and use morphological features that have little variation within a single kidney, while reflecting inter-donor differences indicative of kidney health. Secondly, biopsy quality criteria such as minimum required surface area that control the measurement uncertainty need to be defined and followed. Thirdly, development of normalisation methods to account for variations in biopsy processing is needed to improve score reliability and interpretability.

We hope that this study will help practitioners understand the sources of uncertainty in renal biopsy assessment and drive further work on improving the diagnostic utility of renal biopsy.

## Data Availability

The data analysed in this study is subject to the following licenses/restrictions: Permission needs to be obtained for sharing. Requests to access these datasets should be directed to JA, tobi.ayorinde@nhs.net; XL, xavier.loizeau@npl.co.uk.
