# Supplementary material for: Quantifying measurement uncertainty in renal transplant biopsy assessment
Source: Front Nephrol. 2024 Oct 15;4:1458491. doi: 10.3389/fneph.2024.1458491 (PMC11519413; doi:10.3389/fneph.2024.1458491)
Supplement: Supplementary file 1 [file SupplementaryFile1.pdf]

Quantifying measurement uncertainty in renal transplant biopsy assessment - supplementary material

Xavier Loizeau [1,*], Marina Romanchikova [1], Spencer A. Thomas [1], Moulham Alsuleman [1], John O. O. Ayorinde [2], Gavin J. Pettigrew [2]

[1]National Physical Laboratory, Hampton Road, Teddington, TW11 0LW, United Kingdom
[2]Department of Surgery, University of Cambridge, Addenbrooke's Hospital, Cambridge Biomedical Campus, Cambridge, CB2 0QQ, United Kingdom

Correspondence*:
Xavier Loizeau
xavier.loizeau@npl.co.uk

1 CALCULATION

1.1 Analysis of sensitivity of morphological variables to change in kidney health

Consider a morphological variable Y (e.g., glomeruli per unit area). Denote by M the number of donors, and for donor i, denote by N_i the number of observations for Y from the ith donor, so $N = \sum_{i=1}^{M} N_i$ is the total number of observations for Y. Let Y_i be the vector of observations of Y in the ith donor and $Y_{i,j}$ the jth observation of Y in the ith donor.

Mean of a variable: the average measured value of Y for the entire population, defined by:

$$\mathbb{E}(Y) = N^{-1} \sum_{i=1}^{M} \sum_{j=1}^{N_i} Y_{i,j}. \tag{1}$$

Variance of a variable: a measure of dispersion of a variable around its mean value defined by:

$$\mathbb{V}(Y) = N^{-1} \sum_{i=1}^{M} \sum_{j=1}^{N_i} \left[Y_{i,j} - \mathbb{E}(Y)\right]^2. \tag{2}$$

Intra-donor mean: the average value of Y for a given donor defined by:

$$\mathbb{E}\left(Y|\text{Donor} = i\right) = N_i^{-1} \sum_{j=1}^{N_i} Y_{i,j}. \tag{3}$$

Intra-donor variance: the variance of Y for a given donor (that is, the dispersion of the measured values around the mean measured value for this donor):

$$\mathbb{V}\left(Y|\text{Donor} = i\right) = N_i^{-1} \sum_{j=1}^{N_i} \left[Y_{i,j} - \mathbb{E}\left(Y|\text{Donor} = i\right)\right]^2. \tag{4}$$

This notion is extended to the entire population by taking the mean over all donors of the intra-donor variances, that is, the average dispersion of the measured values around the mean measured values:

$$\mathbb{E}\left[\mathbb{V}\left(Y|\text{Donor}=i\right)\right] = N^{-1}\sum_{i=1}^{M} N_i \mathbb{V}\left(Y|\text{Donor}=i\right). \quad (5)$$

Inter-donor variance: the dispersion of the population of mean values for each donor:

$$\mathbb{V}\left[\mathbb{E}\left(Y|\text{Donor}=i\right)\right] = N^{-1}\sum_{i=1}^{M} N_i \left[\mathbb{E}\left(Y|\text{Donor}=i\right) - \mathbb{E}(Y)\right]^2. \quad (6)$$

Proportion of explained variance: as the total population variance can be decomposed between its inter and intra donor variance in the following way (law of total variance Weiss et al. (2006)):

$$\mathbb{V}(Y) = \mathbb{E}\left[\mathbb{V}\left(Y|\text{Donor}=i\right)\right] + \mathbb{V}\left[\mathbb{E}\left(Y|\text{Donor}=i\right)\right], \quad (7)$$

the proportion of variance explained by the difference of health between donors can be written as:

$$0 \leq \frac{\mathbb{V}\left[\mathbb{E}\left(Y|\text{Donor}=i\right)\right]}{\mathbb{V}(Y)} \leq 1. \quad (8)$$

1.2 Intra-kidney ranking

Table 1 illustrates the resolution of ranking ties for intra-kidney observations. In kidney 1, observations 1 and 3 share the same rank. This tie is resolved by assigning an average rank of two adjacent ranks, resulting in the rank $(1+2)/2$. In Kidney 2 the observations are ranked in ascending order. In kidney 3, all three ranks are identical, and share the average rank of $(1+2+3)/3$.

Kidney ID	Glomeruli count	Rank
1	15	1.5
1	20	3
1	15	1.5
2	30	3
2	15	2
2	10	1
3	20	2
3	20	2
3	20	2

Table 1. Working example of intra-kidney rank calculation for repeated measurements of the variable Glomeruli count.

1.3 Defining an adequacy criterion for glomeruli sub-score

The uncertainty in the measurement of the fraction of sclerotic or healthy glomeruli decreases with an increase in the glomeruli statistics (total number of glomeruli). This rational applies to other intensive and extensive morphometric variables. Below we give a statistical perspective on a glomeruli adequacy criterion to guarantee the validity of the glomeruli sub-score for a well-defined hypothesis.

Uniformity assumption: the proportion of sclerotic glomeruli is uniform across the entire cortex.

Goal: under the uniformity assumption, define a glomeruli adequacy criterion that ensures that the biopsy enables to distinguish between two levels of glomeruli health with a quantified confidence. In statistical terms, it should allow testing whether the proportion of sclerotic glomeruli p, is indeed smaller or larger than the selected threshold value $p^\star$.

Implementation: testing for $\mathbb{H}_0 : p > p^\star; \mathbb{H}_1 : p \leq p^\star$ (null hypothesis: the true proportion of sclerotic glomeruli in the kidney is larger than the threshold value $p^\star$, alternative hypothesis: the true proportion of sclerotic glomeruli in the kidney is smaller than or equal to the threshold value $p^\star$) is done through a binomial test. Hence, if one wants to control the risk of wrongly rejecting $\mathbb{H}_0$ (assuming the kidney quality is sufficient when it is not) to a level $\alpha \in (0, 1)$, one should reject it if the observed proportion of sclerotic glomeruli $\widetilde{p}$ is smaller than the α quantile of a binomial distribution with parameters n and $p^\star$ where n is the number of observed glomeruli ($\widetilde{p} \leq b_{n,p^\star}(\alpha)/n$). Note that the closer p is to $p^\star$, the more difficult is the test, and the larger n is, the easier is the test. For chosen α and tolerance ϵ on $p^\star$, one can determine the minimal number of glomeruli $n^\star$ such that the probability to accept $\mathbb{H}_0$ wrongly (assuming kidney quality is not sufficient when it is) is controlled to a chosen level $\beta \in (0, 1)$. Namely,

$$\begin{aligned} n^\star &= \min \left\{ n \in \mathbb{N} : \mathbb{P}\left(n\widetilde{p} \geq b_{n,p^\star}(\alpha) | p \leq (1-\epsilon)p^\star\right) \leq \beta \right\} \\ &= \min \left\{ n \in \mathbb{N} : \sum_{k=b_{n,p^\star}(\alpha)}^{n} \binom{n}{k} \left[(1-\epsilon)p^\star\right]^k \left[1-(1-\epsilon)p^\star\right]^{n-k} \leq \beta \right\}. \end{aligned} \tag{9}$$

From this definition, $n^\star$ can be determined numerically from values of $\alpha, \beta, p^\star$, and ϵ. One should note that for any value of α, β, and ϵ the value of $p^\star$ leading to the largest value of $n^\star$ is $p^\star = 0.50$. The minimal number of glomeruli needed to control Type 1 and Type 2 errors below 15 % and 20 % respectively for different values of ϵ is represented in fig. 1.

Recommendation for target tissue surface: if the criterion given by the adequacy score is considered sufficient, one can consider the distribution of the number of glomeruli per unit area and try to minimise the biopsy volume while ensuring the adequacy of the biopsy provided with high probability. Only the biopsies without medulla (cortex-only biopsies) are considered. As observed in fig. 2 when the medulla is not present (e.g., in cortex-only biopsies), 90 % of the biopsies have a concentration in glomeruli $C \geq 1\,\text{glomeruli} \cdot \mu\text{m}^{-2}$. Hence, to ensure a 90 % probability of generating an adequate biopsy, one should aim to make its area at least $n^\star/C\,\mu\text{m}^2$.

REFERENCES

Weiss, N. A., Holmes, P. T., and Hardy, M. (2006). A Course in Probability

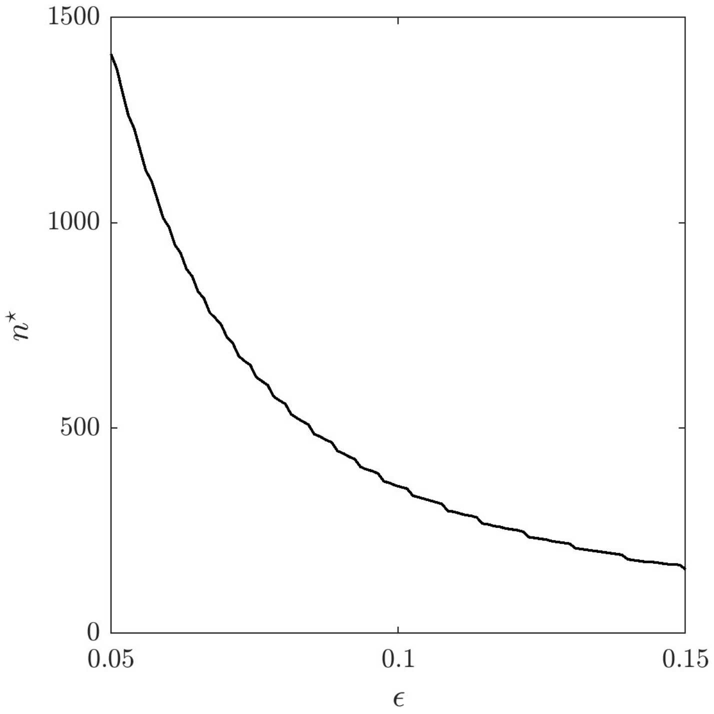

Figure 1. Minimal number of glomeruli $n^\star$ required to achieve a probability of $\leq 0.15 (\leq 0.2)$ of under-/over-estimating the percentage GS.

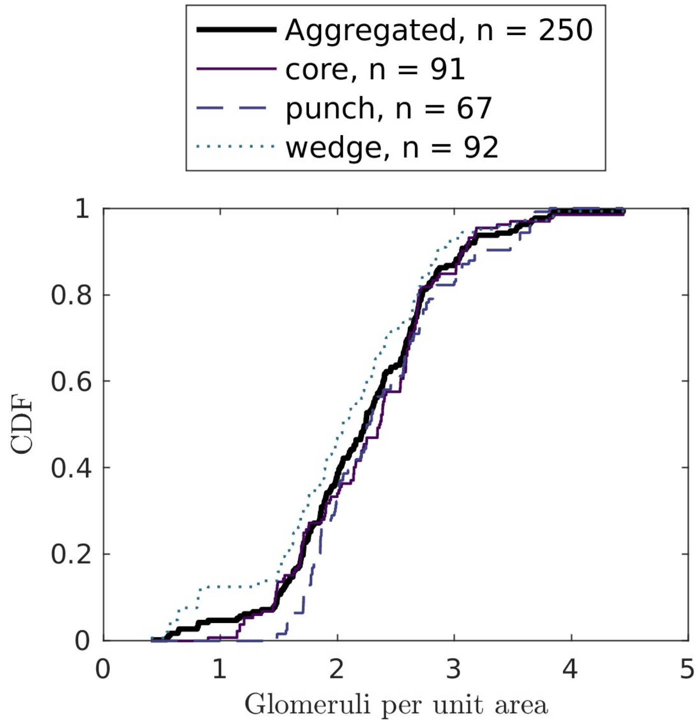

Figure 2. Cumulative distribution functions of glomeruli counts per unit area (in μm^{-2}) by biopsy technique. Only the biopsies without medulla (cortex-only biopsies) are considered (reproduction of Figure 5 in the main text).

Variables and units of measurement	Intensive/Extensive	Category
Biopsy date	-	Biopsy processing
Surgeon anonymised ID	-	Biopsy processing
Staining type (H&E / PAS)	-	Biopsy processing
Biopsy technique (core / punch / wedge)	-	Biopsy processing
Donor anonymised ID	-	Donor
Donor state (brain dead / no survival perspective)	-	Donor
Cause of death (hypoxic brain injury / intracranial even)	-	Donor
Age (years)	-	Donor
Sex (female / male)	-	Donor
Blood type (A / B / AB / O)	-	Donor
Body-mass index ($kg \cdot m^{-2}$)	-	Donor
Donor terminal creatinine ($\mu mol \cdot L^{-1}$)	-	Donor
Estimated amount of blood filtered ($mL \cdot min^{-1} \cdot 0\,33$)	-	Donor
Hypertension (True / False)	-	Donor
Diabetes mellitus (True / False)	-	Donor
Smoker (True / False)	-	Donor
Technical issues (True / False)	-	Donor
Depth of the biopsy (mm)	Extensive	Morphological
Width of the biopsy (mm)	Extensive	Morphological
Total cortex area on image (μm^2)	Extensive	Morphological
Presence of medulla (True / False)	-	Morphological
Large artery puncture (True / False)	-	Morphological
Glomeruli count (count)	Extensive	Morphological
Sclerotic glomeruli count (count)	Extensive	Morphological
Artery count (count)	Extensive	Morphological
Arteriole count (count)	Extensive	Morphological
Diameter of artery (mm)	Intensive	Morphological
Lumen of artery (mm)	Intensive	Morphological
Thickness of artery wall (mm)	Intensive	Morphological
Diameter of 2nd worst artery (mm)	Intensive	Morphological
Lumen of 2nd worst artery (mm)	Intensive	Morphological
Thickness of 2nd worst artery wall (mm)	Intensive	Morphological
Diameter of arteriole (mm)	Intensive	Morphological
Lumen of arteriole (mm)	Intensive	Morphological
Thickness of arteriole wall(mm)	Intensive	Morphological
Area of IF (μm^2)	Extensive	Morphological
Area of TA (μm^2)	Extensive	Morphological

Table 2. Clinical and morphological variables in the original dataset. Morphological variables are categorised into extensive (area-dependent) and intensive (independent of tissue area), where applicable.

Variable	Value	Proportion (%)	Average (range)
Donor state	Brain dead	16.7	–
	No survival perspective	83.3	–
Cause of death	Hypoxic brain injury	53	–
	Intracranial event	47	–
Sex	Female	54	–
	Male	46	–
Comorbidity	Age (years)	–	65.4 ($[52, 76]$)
	Diabetes	8	–
	Hypertension	68	–
	Smoking	49	–
	Body mass index	–	28.7 ($[16.5, 30.5]$)
	Terminal creatinine ($\mu mol \cdot L^{-1}$)	–	73.2 ($[46, 138]$)

Table 3. Clinical characteristics of the study cohort.

Presence	Remuzzi and morphometric variables	Biopsy processing variable	p-value
Observed	Depth of the biopsy	Biopsy technique	$< 10^{-3}$
Observed	Width of the biopsy	Biopsy technique	$< 10^{-3}$
Observed	Total area of the biopsy	Biopsy technique	$< 10^{-3}$
Observed	Glomeruli count	Biopsy technique	$< 10^{-3}$
Observed	Healthy glomeruli count	Biopsy technique	$< 10^{-3}$
Observed	Area of IF	Biopsy technique	$< 10^{-3}$
Observed	Area of TA	Biopsy technique	$< 10^{-3}$
Observed	Percentage of TA	Stain type	$< 10^{-3}$
Observed	Percentage of IF	Stain type	$< 10^{-3}$
Observed	Sclerotic glomeruli count	Biopsy technique	$< 10^{-3}$
Observed	Area of TA	Stain type	$< 10^{-3}$
Observed	Remuzzi score, TA	Stain type	$< 10^{-3}$
Observed	Remuzzi score, IF	Stain type	$< 10^{-3}$
Observed	Area of IF	Stain type	$< 10^{-3}$
Observed	Total Remuzzi score	Stain type	$< 10^{-3}$
Observed	Transplant decision	Stain type	$< 10^{-3}$
Missing	Area of IF	Stain type	$< 10^{-3}$
Missing	Area of TA	Stain type	$< 10^{-3}$

Table 4. All dependency tests where the null hypothesis is rejected, i.e., the processing variable influenced the morphological variable. The ”missing” column states whether the effect is on the risk of missing values or on the value when observed.

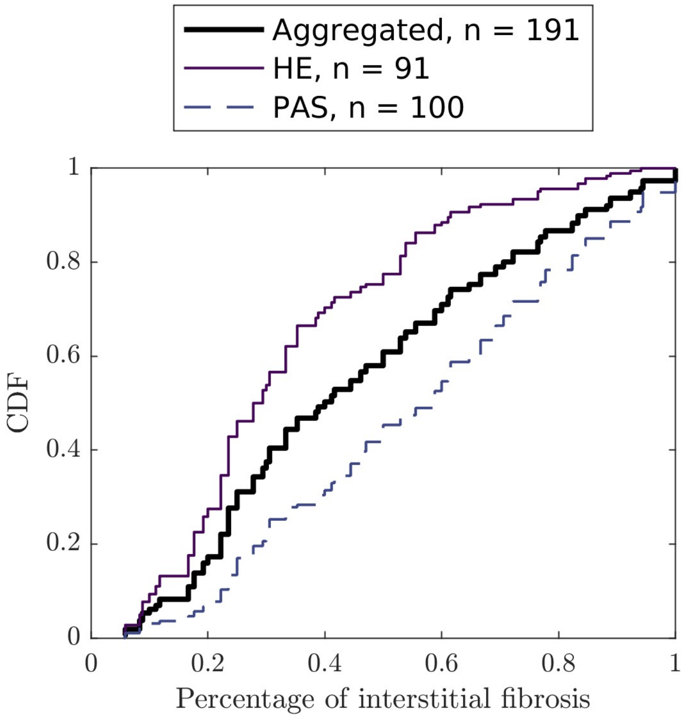

Figure 3. Cumulative Distribution Function of the ranked percentage of interstitial fibrosis grouped by staining technique. p-value < 0.001.

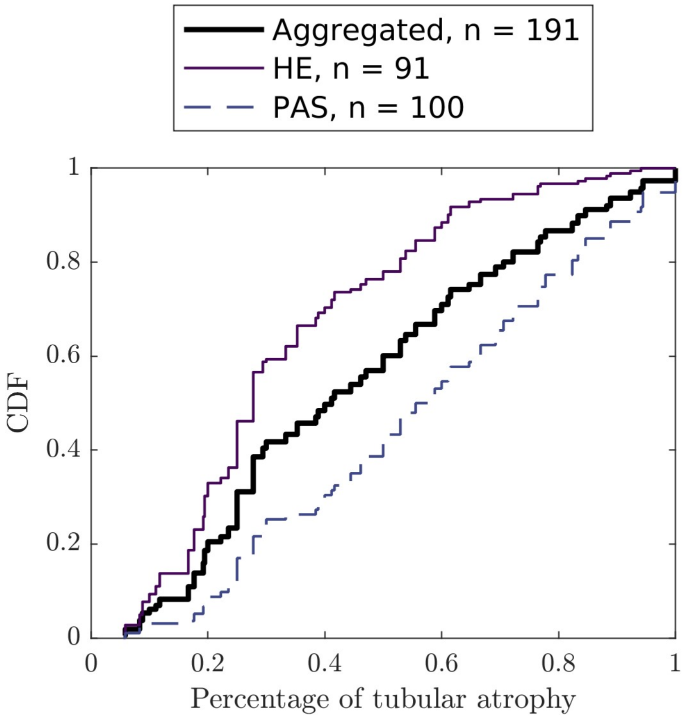

Figure 4. Cumulative Distribution Function of the ranked percentage of tubular atrophy grouped by staining technique. p-value < 0.001.
